# Experimental Evaluation of the Pathogenicity of Different Strains of *Schistosoma mansoni*

**DOI:** 10.1155/2012/894940

**Published:** 2012-11-07

**Authors:** Antônio Aurélio Euzébio, Nádia Regina Borim Zuim, Arício Xavier Linhares, Luiz Augusto Magalhães, Eliana Maria Zanotti-Magalhães

**Affiliations:** ^1^Department of Pediatrics, School of Medical Sciences, State University of Campinas (Unicamp), Rua Tessália Vieira de Camargo, 126 Cidade Universitária Zeferino Vaz, 13083-887 Campinas, SP, Brazil; ^2^Department of Animal Biology, Institute of Biology, State University of Campinas (Unicamp), Rua Monteiro Lobato, 255 Cidade Universitária Zeferino Vaz, 13083-862 Campinas, SP, Brazil

## Abstract

The pathogenesis of three different *Schistosoma mansoni* strains from the Brazilian states of Minas Gerais (BH strain) and São Paulo (SJ and SD strains) was evaluated in experimentally infected mice. Observations of the most severe clinical cases among local patients treated (SD strain) in the city of Campinas (São Paulo, Brazil) formed the basis of this study. Mice were used as definitive hosts and were infected with cercariae from *Biomphalaria tenagophila* (SJ and SD strains) and *Biomphalaria glabrata* (BH strains). The parameters analyzed were as follows: number of *S. mansoni* eggs in mice feces; number of granulomas per tissue area in liver, spleen, lungs, pancreas, and ascending colon; measurements of hepatic and intestinal granulomas; number of adult worms; and measurements of trematode eggs. The comparison among the three strains indicated that the SD strain, isolated in Campinas, presented a higher worm recovery relative to the number of penetrating cercariae. In addition, when compared to the SJ and BH strains, the SD strain demonstrated similar pathogenicity to the BH strain, with a greater quantity of granulomas in the viscera, as well as larger granulomas and eggs. Furthermore, a greater quantity of trematode eggs was also shed in the feces.

## 1. Introduction

 After malaria, schistosomiasis is the most important global parasitic disease and is a serious public health problem mainly in tropical and subtropical regions. In Brazil, the spread of schistosomiasis is caused by the single species occurring in the country, *Schistosoma mansoni*. It has been shown that the spread of *S. mansoni* is directly related to the economic history of the country via human migratory events from hyperendemic regions and the presence of three species of snails, which are the natural intermediate hosts of the parasite. Schistosomiasis, initially described as a typically rural disease, now has an urban character and is prevalent mainly on the outskirts of large cities in São Paulo (Brazil), the most populous state in Brazil. Another feature of the disease in São Paulo state is the absence of severe cases [1], which is favorable in one sense; however, due to the absence of clinical manifestations that bring patients to the public health clinics, schistosomiasis often remains undiagnosed, and the infected individuals may continue to contaminate fresh water sources where the intermediate hosts breed.

In 1963 [2], the BH and SJ strains of *Schistosoma mansoni* were initially described based on the observation that *Biomphalaria glabrata* from Belo Horizonte (in the state of Minas Gerais, Brazil) could be infected with a local strain (BH) of *S. mansoni* but was resistant to infection by *S. mansoni *isolated from the Vale do Rio Paraiba do Sul (the South Paraiba River Valley) (SJ strain) from São Paulo, Brazil. Moreover, *Biomphalaria tenagophila* from the Vale do Rio Paraíba do Sul was susceptible to the local *S. mansoni* (SJ strain) but refractory to infection by *S. mansoni* from Belo Horizonte (BH strain).

Studies in mice [3, 4] revealed morphological differences between the worms of these two strains as well as differences in their pathogenicity [5–7]. The authors [7] concluded that a much smaller number of BH-strain parasites lead to equivalent levels of damage caused by greater numbers of SJ-strain worms.

In BH-strain endemic areas, individuals with decompensated hepatosplenomegaly are commonly observed. In areas where patients are infected with the SJ strain, the commonly described clinical manifestations include the intestinal form with a slightly enlarged spleen [8].

In Campinas, a city located in the state of São Paulo (Brazil) with a population of approximately one million, the first native case of schistosomiasis from *S. mansoni* was observed in 1959, according to the records of the Health Secretariat of São Paulo, and originated from a focus at the Piçarrão stream [9]. Since then, new cases and new infection foci have been recorded in Campinas and its surrounding regions. However, most of the cases have been asymptomatic.

Autochthonous cases of schistosomiasis from *S. mansoni* have been reported in the Jardim São Domingos (São Domingos Garden) neighborhood of Campinas since 1998. More recently, a strain of the trematode was isolated in a laboratory from* B. tenagophila* captured at Lagoa Boa União (Boa União Lake), which is located in the Jardim São Domingos neighborhood. Freitas and Oliveira [10] and Freitas et al. [11] found signs of portal hypertension and, in some cases, myelopathy in patients with autochthonous schistosomiasis treated at the Jardim São Domingos Medical Center. These facts led us to conduct comparative studies of the pathogenicity of different *S. mansoni *strains isolated from Jardim São Domingos (Campinas, São Paulo, Brazil), BH strains from Belo Horizonte (Minas Gerais, Brazil), and SJ strains from the Vale do Rio Paraíba do Sul (São Paulo, Brazil).

## 2. Materials and Methods

The experiments were approved by the Animal Experimentation Ethics Committee from our institution and were certified under protocol number 642-1.

Swiss specific pathogen free (SPF) female mice were exposed to infection by *S. mansoni* cercariae of BH strains (originally from Belo Horizonte, Minas Gerais, Brazil), SJ strains (originally from the Vale do Rio Paraíba, São Paulo, Brazil), and SD strains (originally from Jardim São Domingos, Campinas, São Paulo, Brazil). The mice were distributed into three groups: Group 1, mice infected with *S. mansoni* cercariae of the BH strain; Group 2, mice infected with *S. mansoni* cercariae of the SJ strain; and Group 3, mice infected with *S. mansoni* cercariae of the SD strain.

Starting at the third week of infection, mice fecal matter was collected weekly to verify the elimination of *S. mansoni* eggs. At the eighth week of infection, all surviving mice were sacrificed for worm recovery.

The evaluated parameters included the following: the number of penetrating cercariae; the number of eggs shed in feces; the number of worms in the mesenteric-portal system on the day the mice were sacrificed; the number of granulomas in the liver, spleen, intestine (ascending colon), pancreas, and lung; tissue area of granulomas found in the liver and intestine; and *S. mansoni *egg size.

### 2.1. Infection of Mice

Mice were infected with *S. mansoni *cercariae from sympatric snails bred in the laboratory from *B. tenagophila* captured at Lagoa Boa União, Jardim São Domingos (23°02′21.31′′S 47°06′17.01′′W) in Campinas. These snails were exposed to miracidia of the SD strain. The *B. glabrata* from Belo Horizonte was exposed to miracidia of the BH strain, and the *B. tenagophila* from the Vale do Rio Paraiba do Sul was exposed to miracidia of the SJ strain.

Mice were individually exposed for two hours to cercariae from the snails infected with different strains. Infection was performed percutaneously by exposure of the tail to 70 cercariae, according to the method described by Magalhães [12].

### 2.2. Checking the Number of *S. mansoni* Eggs Shed in the Feces

The number of eggs eliminated in the feces of infected mice was determined using the method described by Kato-Katz starting from the third week of infection [13]. Glass slides were prepared for each group of five animals, and the final report results represent the arithmetic means of the readings from all glass slides.

### 2.3. Collection and Counting of Worms

Animals were sacrificed by cervical dislocation at the eighth week of infection. Worms were collected through the perfusion of the hepatic portal system according to the method described by Yolles et al. [14]. The number of male and female worms was recorded, whether they were isolates or pairs.

### 2.4. Collection, Counting, and Measurement of Schistosomal Granulomas of the Viscera

After perfusion, fragments of the liver, spleen, colon, lung, and pancreas of the sacrificed animals were extracted for granuloma counting and measurement. The fragments were fixed in aqueous Bouin's solution, and 5 *μ*m thick histological sections were stained with Masson's trichrome stain. The sections were analyzed with an optical microscope to calculate the number of granulomas per tissue area (1.2469 mm^2^) based on the method described by Magalhães et al. [6]. The sizes of the granulomatous reactions were determined based on their area. Only granulomas containing an *S. mansoni* egg in their center were measured, as the egg verified the proximity of the sectioning to larger granulomatous reactions. The measurements were performed using the Image Pro Lite software, version 4.0 for Windows 95/NT/98.

### 2.5. Collection and Measurement of *S. mansoni *Eggs

Eggs of the BH, SJ, and SD strains of *S. mansoni* were collected from the feces of infected mice. The length, width, and length of the egg spicules were measured using Image Pro Lite, version 4.0 for Windows 95/NT/98. Statistical analysis of the data included examination of the length, width, spicule size, length to width ratio, length to spicule ratio, and width to spicule ratio. The PROC GLM (General Linear Procedure) function in the SAS software package (2006) was used for the analysis [15].

## 3. Results

All animals infected with all three strains of *S. mansoni* survived for the eight weeks of the experiment.

Tables 1 and 2 list the mean numbers of penetrating cercariae, male worms, female worms, mating worm pairs, total number of worms, and percentage of worms recovered relative to the number of penetrating cercariae in mice infected with the BH, SJ, and SD strains of *S. mansoni*.

Statistical analysis indicated that the penetration capacity of the cercariae in mice was significantly different among the strains (*P* = 0.0241). The SJ cercariae presented greater penetration than the BH cercariae (Table 1).

The number of mating worm pairs and the total number of worms were significantly different among the strains (*P* = 0.0020 and *P* = 0.0036, resp.). The mice infected with the SD strain had a significantly higher number of worms (mating pairs and total) than the mice infected with the BH strain (Table 2).

The percentage of recovered worms relative to the number of penetrating cercariae was significantly different by strain (*P* = 0.0065). As listed in Table 2, the worm recovery percentage was higher from mice infected with the SD strain. Statistical analysis (Table 1) also revealed that the numbers of male worms and female worms recovered from mice did not differ significantly among all three strains (*P* = 0.7389 and *P* = 0.4560, resp.).

Table 3 lists the number of granulomatous reactions around the *S. mansoni* eggs per tissue area (1.2469 mm^2^) found in the viscera of mice infected with the BH, SJ, and SD strains of *S. mansoni*.

The mean number of granulomatous reactions found in the liver was significantly different (*P* = 0.0032) by strain. Mice infected with the BH and SD strains exhibited a greater number of granulomatous reactions compared to those infected with the SJ strain (Table 3).

There was a significant difference in the mean number of splenic granulomas in mice infected with different strains (*P* = 0.0390), and the SD strain produced a higher number of granulomas compared to the BH and SJ strains. The difference between the values for the BH and SJ strains was not significant (Table 3).

Histological analysis of the pancreas sections revealed a greater mean number of schistosomal granulomas for the SD strain (Table 3) compared to the other strains (*P* = 0.0189).

Statistical analysis of the number of granulomas in the intestine (ascending colon) and lung did not reveal any significant difference among all three strains (*P* = 0.2134 and *P* = 0.2473, resp.). However, the SD strain produced a greater mean number of granulomas in the intestines. In addition, the SD strain did not produce a greater number of granulomas in the lung than the BH strain (Table 3).

In general, the histological analysis of sections from mice infected with all three strains indicated that the liver had the greatest number of granulomatous reactions around the *S. mansoni *eggs, followed by the intestines. The third-greatest number of granulomas for the SD strain was found in the pancreas, followed by the spleen and lungs. For the BH strain, the pancreas also had the third-greatest number of visceral granulomas; however, unlike the SD strain, the pancreas was followed by the lungs and then the spleen. For the SJ strain, similar numbers of granulomas were found in the pancreas and spleen. No granulomas were observed in the lungs of animals infected with the SJ strain.

The values obtained for the average size of granulomatous reactions and the number of eggs per gram of feces are presented for each strain in Tables 4 and 5.

The data in Table 4 indicate that the largest granulomatous area in the liver was found for the SD strain. The hepatic granulomas in mice infected with the BH and SJ strains were smaller and did not differ significantly from each other. 

The intestinal granulomas were larger in animals infected with the SD and BH strains, followed by those infected with the SJ strain (Table 4).

By the seventh and eighth weeks of infection, it was evident that mice infected with the SD strain tended to eliminate similar numbers of eggs to the BH strain (Table 5), despite having *B. tenagophila* as an intermediate host. However, it is important to note that the total worm number and mating worm pair recovery in the mesenteric portal system of mice infected with the SD strain was greater on average than in mice infected with the BH strain (Table 2).

Table 6 lists egg measurement data. The length and width of the SD-strain eggs (Table 6) were significantly longer than the other eggs from other strains (*P* < 0.0001 and *P* < 0.0001, resp.), and the eggs of the BH and SJ strains did not differ in length. Eggs from the BH strain had the shortest width.

**Figure 1:**
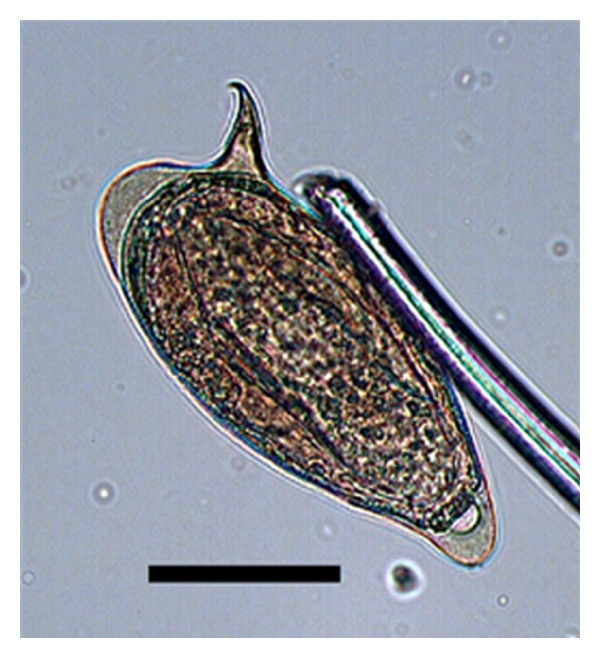
Egg from the SD strain of *S. mansoni* (bar = 50 *μ*m) observed in the feces of mice. Note the curved spicule tip.

The size of the spicule (Table 6) was not significantly different among the strains (*P* = 0.9383); however, some eggs from the SD strain had spicules with a very curved tip, as shown in Figure 1. The measurement ratio data in Table 7 indicate that the strains differed only in the width to spicule length ratio (*P* = 0.0855), which was highest for the SD strain.

## 4. Discussion

 Geographically isolated strains of *S. mansoni* present significant differences in pathogenicity, which is attributed to greater impairment of organs due to the greater extent and wider distribution of *S. mansoni *eggs [16], the number of eggs produced by the parasite [7, 17], and the degree of susceptibility of the snail vector [1, 16, 18, 19]. According to Chieffi and Waldman [1], the rarity of hepatosplenic forms in autochthonous cases in São Paulo State (Brazil) may be due to the low rates of snail infection by *S. mansoni*, which reflects the lower susceptibility of infection of *B. tenagophila* compared to *B. glabrata* and *B. straminea*.

In a previous study, Yoshioka et al. [20] demonstrated that the Santa Rosa (SR) strain of *S. mansoni* isolated in Campinas, São Paulo State (Brazil), was less pathogenic than the BH and SJ strains. Mice infected with the SR strain exhibited the fewest worms, eggs shed in the feces, and granulomas. The parasite eggs also had the smallest diameter of granulomatous reaction around them. 

Bina and Prata [17] have shown an association between the development of the hepatosplenic disease form and the quantity of *S. mansoni* eggs eliminated in patient stools. The lesions caused by *S. mansoni* are rarely restricted to one organ, whether it is the liver or intestine. *S. mansoni* eggs are usually found in almost all organs and tissues, where they produce some similar reactions. Certain differences arise from the specific characteristics of the affected tissues. 

 In the studied strains, schistosomal granulomas were found in all studied organs, except for the lungs of mice infected with the SJ strain (Table 3).

The results presented here indicate high pathogenicity for the São Domingos strain (SD), which was initially isolated from the São Domingos neighborhood in Campinas (São Paulo state). This strain had similar levels of pathogenicity to the BH strain, when considering the number and size of granulomatous reactions in the viscera. The greater involvement of tissues and organs can be attributed to the greater number of eggs shed by the worms and to the larger egg size of the SD strain. In addition to being more numerous, the eggs of the SD strain were larger (Table 6) and had a distinct morphological feature, a curved spicule tip (Figure 1).

From an epidemiological point of view, these results are relevant due to the greater worm recovery relative to the number of penetrating cercariae and higher number of eggs shed in the feces of animals infected with the SD strain.

The data obtained for the SD strain of *S. mansoni* also indicate that this strain is the most pathogenic of all strains currently described that have *B. tenagophila* as an intermediate host. The greater pathogenicity of the SD strain observed experimentally in laboratory animals confirms the observations obtained from clinical examinations of patients who were infected while bathing in areas contaminated with *B. tenagophila* [10, 11]. Freitas and Oliveira [10] have observed myelopathy in some of these patients. Myelopathy is considered one of the most serious problems caused by *Schistosoma mansoni* infection, and all evidence suggests that parasite eggs are responsible for its clinical manifestation [21].

The results of the present study seem to indicate that the more severe clinical forms of *S. mansoni*-related schistosomiasis in immunologically competent individuals may be caused by higher parasite load, higher fecundity of the worms, and greater numbers of eggs. All of these lead to a more severe compromise of tissues due to a greater number of schistosomal granulomas. The severity of the disease may also be attributed to the larger size of the trematode eggs, which generate more antigens in the host organism, thereby raising the host's sensitivity and promoting inflammation, which, in turn, produces a greater extent of tissue damage.

Based on the data presented in this study, an accurate evaluation of *S. mansoni* pathogenicity has to be based on several biological characteristics of the parasite: the immunoregulatory role involved in the granulomatous response, the trematode egg antigens responsible for inducing the granuloma, the genetic peculiarities of the host [22], and the parasite load in the clinical manifestation of schistosomiasis in mice and humans.

## Figures and Tables

**Table 1:** Mean numbers of penetrating cercariae and mean numbers of male and female worms in mice infected with BH, SJ, and SD strains of* S. mansoni* and sacrificed at the eighth week of infection.

*S*. *mansoni* strain	Number of infected mice	Penetrating cercariae	Male worms	Female worms	Duncan's Test*	
SJ	10	66.90			A	
SD	10	64.50			A	B
BH	15	62.67				B

BH	15		1.73		A	
SD	10		1.30		A	
SJ	10		0.90		A	

SJ	10			0.30	A	
BH	15			0.13	A	
SD	10			0.10	A	

*Means followed by same letter do not differ significantly from each other (*α* = 0.05).

**Table 2:** Mean numbers of mating worm pairs, total number of worms, and mean percentages of worm recovery relative to the number of penetrating cercariae in mice infected with BH, SJ, and SD strains of* S. mansoni* and sacrificed at the eighth week of infection.

*S. mansoni* strain	Number of infected mice	Mating worm pairs	Total number of worms	Worm recovery (%)	Duncan's test*	
SD	10	14.80			A	
SJ	10	11.60			A	B
BH	15	7.87				B

SD	10		31.00		A	
SJ	10		24.40		A	B
BH	15		17.60			B

SD	10			48.62	A	
SJ	10			36.67	A	B
BH	15			28.47		B

*Means followed by same letter do not differ significantly from each other (*α* = 0.05).

**Table 3:** Mean numbers of granulomas per tissue area (tissue area of 1.2469 mm^2^) in mice infected with the BH, SJ, and SD strains of *S. mansoni* and sacrificed at the eighth week of infection.

*S. mansoni* strain	Number of mice	Number of granulomas (mean)/1.2469 mm^2^						
		Liver	Spleen	Pancreas	Intestines	Lungs		Duncan's test*
BH	15	15.27					A	
SD	10	13.80					A	
SJ	10	10.60						B
SD	10		1.00				A	
BH	15		0.13					B
SJ	10		0.10					B
SD	10			3.40			A	
BH	15			2.00			A	B
SJ	10			0.10				B
SD	10				4.50		A	
BH	15				3.87			
SJ	10				2.50		A	
BH	15					1.07	A	
SD	10					0.40	A	
SJ	10					0.00	A	

*Means followed by same letter do not differ significantly from each other (*α* = 0.05).

**Table 4:** Mean areas of the hepatic and intestinal granulomas at the eighth week of infection in mice infected with BH, SJ, and SD strains of* S. mansoni*.

*S.mansoni* strain	Number of granulomas	Granuloma area (mean) *μ*m^2^		Duncan's test*
		Hepatic	Intestinal	
SD	206	132194		A
BH	403	111586		B
SJ	137	109472		B
SD	32		78313	A
BH	41		72340	A
SJ	16		38667	B

*Means followed by same letter do not differ significantly from each other (*α* = 0.05).

**Table 5:** Mean numbers (log) of *S. mansoni* eggs from mice infected with the BH, SJ, and SD strains per gram of feces.

*S. mansoni* strain	Number of infected mice	Number of eggs (log-mean)	Duncan's test*	Infection Time (week)
SJ	10	1.417	A	3rd
BH	15	0.944	A	
SD	10	0.000	A	
SJ	10	3.186	A	4th
BH	15	0.944	A	
SD	10	0.000	A	
SJ	10	4.342	A	5th
SD	10	3.967	A	
BH	15	2.409	B	
SD	10	4.309	A	6th
SJ	10	3.804	A	
BH	15	3.275	A	
SD	10	6.787	A	7th
BH	15	6.579	A	
SJ	10	4.524	B	
SD	10	7.248	A	8th
BH	15	6.746	A	
SJ	10	5.563	B	

*Means followed by same letter do not differ significantly from each other (*α* = 0.05).

**Table 6:** *S. mansoni* egg measurements from the BH, SJ, and SD strains.

*S*. *mansoni* strain	Number of eggs	Egg (mean) *μ*m			Duncan's test*
		Length	Width	Spicule	
SD	23	155.60			A
SJ	31	141.11			B
BH	31	139.34			B
SD	23		64.63		A
SJ	31		59.37		B
BH	31		56.89		C
BH	31			21.22	A
SJ	31			21.09	A
SD	23			20.85	A

*Means followed by same letter do not differ significantly from each other (*α* = 0.05).

**Table 7:** *S. mansoni* egg ratio measurements for the BH, SJ, and SD strains.

*S.mansoni* strain	Number of eggs	Egg (mean)				Duncan's test*
		Length/width	Length/Spicule	Width/spicule	
SD	23	2.45			A	
BH	31	2.41			A	
SJ	31	2.38			A	
SD	23		7.63		A	
BH	31		6.93		A	
SJ	31		6.87		A	
SD	23			3.16	A	
SJ	31			2.89	A	B
BH	31			2.80		B

*Means followed by same letter do not differ significantly from each other (*α* = 0.05).
